# Inflammasome-Mediated IL-1β Production in Humans with Cystic Fibrosis

**DOI:** 10.1371/journal.pone.0037689

**Published:** 2012-05-23

**Authors:** Anthony Tang, Ashish Sharma, Roger Jen, Aaron F. Hirschfeld, Mark A. Chilvers, Pascal M. Lavoie, Stuart E. Turvey

**Affiliations:** 1 Department of Microbiology & Immunology, University of British Columbia, Vancouver, British Columbia, Canada; 2 Department of Experimental Medicine, University of British Columbia, Vancouver, British Columbia, Canada; 3 Department of Pediatrics, British Columbia Children's Hospital and Child & Family Research Institute, Vancouver, British Columbia, Canada; University of California Merced, United States of America

## Abstract

**Background:**

Inflammation and infection are major determinants of disease severity and consequently, the quality of life and outcome for patients with cystic fibrosis (CF). Interleukin-1 beta (IL-1β) is a key inflammatory mediator. Secretion of biologically active IL-1β involves inflammasome-mediated processing. Little is known about the contribution of IL-1β and the inflammasomes in CF inflammatory disease. This study examines inflammasome-mediated IL-1β production in CF bronchial epithelial cell lines and human patients with CF.

**Results:**

Bronchial epithelial cell lines were found to produce negligible amounts of basal or stimulated IL-1β compared to hematopoeitic cells and they did not significantly upregulate caspase-1 activity upon inflammasome stimulation. In contrast, peripheral blood mononuclear cells (PBMCs) from both CF and healthy control subjects produced large amounts of IL-1β and strongly upregulated caspase-1 activity upon inflammasome stimulation. PBMCs from CF patients and controls displayed similar levels of caspase-1 activation and IL-1β production when stimulated with inflammasome activators. This IL-1β production was dependent on NF-κB activity and could be enhanced by priming with LPS. Finally, chemical inhibition of CFTR activity in control PBMCs and THP-1 cells did not significantly alter IL-1β or IL-8 production in response to *P. aeruginosa*.

**Conclusion:**

Hematopoeitic cells appear to be the predominant source of inflammasome-induced pro-inflammatory IL-1β in CF. PBMCs derived from CF subjects display preserved inflammasome activation and IL-1β secretion in response to the major CF pathogen *Pseudomonas aeruginosa*. However, our data do not support the hypothesis that increased IL-1β production in CF subjects is due to an intrinsic increase in NF-κB activity through loss of CFTR function.

## Introduction

Cystic fibrosis is an autosomal recessive disease caused by mutations in the cystic fibrosis transmembrane conductance regulator (CFTR), which functions as a chloride ion channel. CF remains one of the most common lethal genetic diseases in populations of European descent with the current average lifespan of CF patients approximately 40 years of age [1], [2]. Recurrent inflammatory pulmonary exacerbation is the primary cause of lung disease progression and ultimately, death in CF. Controlled inflammation is important for fighting infection, but in excess, it becomes destructive to host cells and to the architecture of the lungs [2], [3]. In CF, airway epithelial cells have been shown to produce an exaggerated pro-inflammatory cytokine response to stimulation [4], [5]. It is unclear whether this heightened inflammatory response is intrinsic to cells lacking CFTR or whether it is a result of chronic polymicrobial infection [6], [7]. Regardless of this controversy, identifying and targeting relevant inflammatory mediators is a critical step in developing more specific therapeutic approaches to control inflammation and improve health outcomes in CF [8].

Interleukin-1 beta (IL-1β) is a major inflammatory mediator. Its physiological effects are diverse and potentially important to the pathogenesis of lung exacerbations in CF, including the generation of fever, the recruitment of inflammatory effector cells, the induction of other pro-inflammatory cytokines such as IL-6 and IL-8, and the shaping of T cell responses [9], [10]. Following initiation of the NF-κB signaling cascade, IL-1β is produced in the cytosol as a biologically inactive full-length pro-IL-1β. Pro-IL-1β is subsequently converted into its active form by cytosolic protein complexes termed “inflammasomes.” Inflammasomes assemble in response to certain cellular danger signals and mediate the auto-activation of caspase-1 [9], [11], which cleaves pro-IL-1β and pro-IL-18 into their biologically active forms for secretion. Four distinct inflammasomes have been recognized. These are the NLRP1 [12], NLRP3 [13], [14], NLRC4 [15], [16], and AIM2 inflammasomes [17], [18], which respond to a variety of different microbial signatures and danger signals [11].

*P. aeruginosa*, one of the most common and clinically relevant pathogens among CF patients, activates the NLRC4 inflammasome [19], [20]. Infection with *P. aeruginosa* triggers an increase in levels of IL-1β, IL-6, and IL-8 in bronchoalveolar lavage fluid (BALF) from patients with CF [21]. Inflammasome responses depend on NF-κB signaling, where NF-κB is important in both the upregulation of specific inflammasome components [22], [23], as well as IL-1β expression [24], [25].

Previous studies support a role for IL-1β in the pathogenesis of CF inflammatory lung disease. Levels of IL-1β are increased in BALF from CF patients with infection [21], [26], [27], [28] and this increase has been temporally associated with a clinical response to treatment [21]. Polymorphisms in the *IL1B* gene have also been associated with varying degrees of disease severity in CF patients [29]. Murine models of CFTR dysfunction have exhibited significant increases in IL-1β expression or secretion in macrophages [30], [31], and support the hypothesis that the loss of CFTR increases NF-κB activation under basal and stimulatory conditions [4], [5], [32], [33]. Finally, replacement of chloride ions with glutamate or gluconate in cell culture media increases secretion of IL-1β in response to NLRP3 stimulation by adenosine triphosphate (ATP) [34], implying an inhibitory role for extracellular chloride in NLRP3 activation. Taken together, these data implicate the involvement of IL-1β and consequently, the inflammasomes, in CF inflammatory disease.

## Results

### Airway epithelial cells do not produce significant amounts of IL-1β in response to inflammasome stimulation

**Figure 1:**
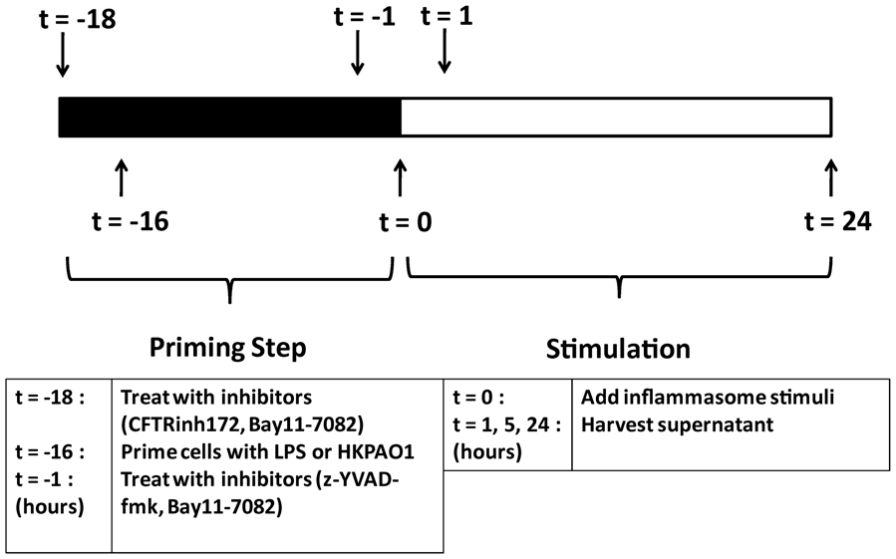
Cell stimulation and inhibitor schedule. Schedule outlines the timing of inhibitor addition and priming in relation to inflammasome stimulation (t = 0) for THP-1 reporter and PBMC cytokine quantification experiments. Inhibitor treatments and stimulations were carried out as described in the Materials and Methods section.

**Figure 2:**
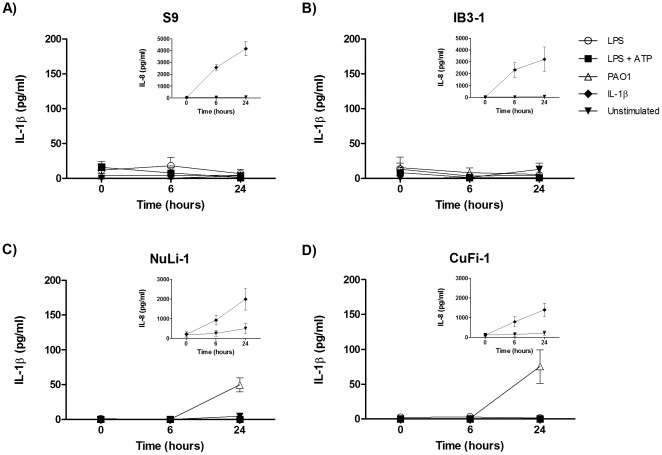
Airway epithelial cells do not significantly contribute to IL-1β production in response to inflammasome stimuli. Control cell lines ((A) S9, (C) NuLi-1) and their corresponding CF cell lines ((B) IB3-1, and (D) CuFi-1) cells were stimulated with *P. aeruginosa* (MOI = 10), ATP (5 mM), or IL-1β (10 ng/ml), for the indicated times (n = 3 individual experiments). Cells were primed with LPS (100 ng/ml) for 4 hours where appropriate. Cell culture supernatants were assayed for IL-1β and IL-8 production by ELISA. Insert shows IL-8 secretion in response to stimulation with IL-1β (10 ng/ml).

The inflammasomes and their respective activators examined in this study are listed in Table 1. Cells were stimulated in accordance with the schedule in Figure 1. In CF, airway epithelial cells have been shown to possess a hyper-inflammatory phenotype and produce an exaggerated pro-inflammatory cytokine response [4], [5]. To determine if airway epithelial cells contribute to the increased IL-1β production in patients with CF, CF and control bronchial epithelial cell lines were stimulated with the inflammasome inducers *P. aeruginosa* strain PAO1 (PAO1) and LPS followed by ATP. IL-1β levels in cell culture supernatants were not greatly increased in either the CF or control cell lines (Fig. 2a–d), although a small increase in IL-1β production was detected in NuLi-1 and CuFi-1 cells, but not in S9 and IB3-1 cells, by 24 hours. In contrast, these airway cells were highly responsive to other inflammatory stimuli, such as recombinant IL-1β, producing large quantities of IL-8 (Fig. 2a–d inserts).

**Table 1:** Inflammasome Activators.

INFLAMMASOME	INFLAMMASOME STIMULUS	EXOGENOUS PRIMING OF NF-κB REQUIRED?
NLRP3	ATP (Concentration: 5 mM)	Yes
NLRC4	*Pseudomonas aeruginosa* strain PAO1. (Multiplicity of Infection (MOI) = 1)	No (primed by live bacterium)
AIM2	Poly(dA:dT) (Concentration: 1 µg/ml)	Yes

### Airway epithelial cells do not significantly upregulate caspase-1 activity in response to inflammasome stimulation

**Figure 3:**
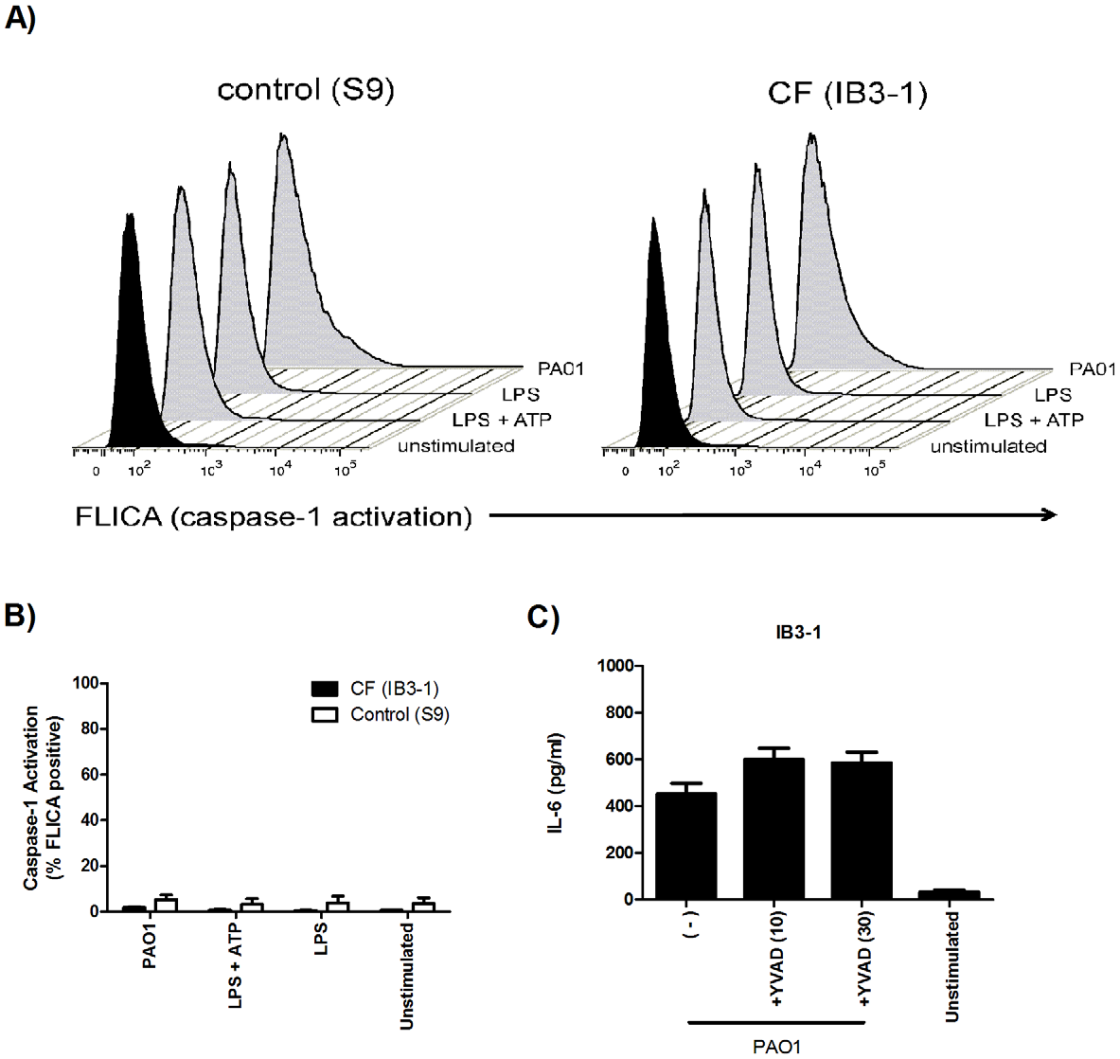
Airway cells do not strongly upregulate caspase-1 activity in response to inflammasome stimuli. S9 and IB3-1 cells were examined for caspase-1 activation following inflammasome stimulation with *P. aeruginosa* (MOI = 10) and ATP (5 mM). Cells were primed with LPS for 5 hours where appropriate. A representative histogram of % caspase-1-active cells is shown in (A) and the averaged values are shown in (B) (n = 3 separate experiments). (C) IB3-1 cells (5×10^4^ cells/well) were pre-treated for 1 hour with increasing concentrations of z-YVAD-fmk (10–30 µM) prior to stimulation with *P. aeruginosa* (MOI = 50). Cell culture supernatants were collected after 6 hours and assayed for IL-6 by ELISA (n = 3).

To examine if inflammasome activation occurs in these airway cells, caspase-1 activity was quantified by flow cytometry. There was no significant increase upon stimulation with live PAO1 or LPS+ATP at the times examined (Fig. 3a–b). Because previous studies have indicated a role for caspase-1 in the activation of NF-κB through Toll-like receptor (TLR) signaling [35], we examined whether chemical inhibition of caspase-1 altered NF-κB-dependent IL-6 production in response to *P. aeruginosa*. However, treatment with the caspase-1 inhibitor z-YVAD-fmk (YVAD) did not decrease IL-6 secretion by airway epithelial cells (Fig. 3c).

### CD14 positive monocytes from CF patients and controls show similar increases in caspase-1 activity upon inflammasome stimulation

**Figure 4:**
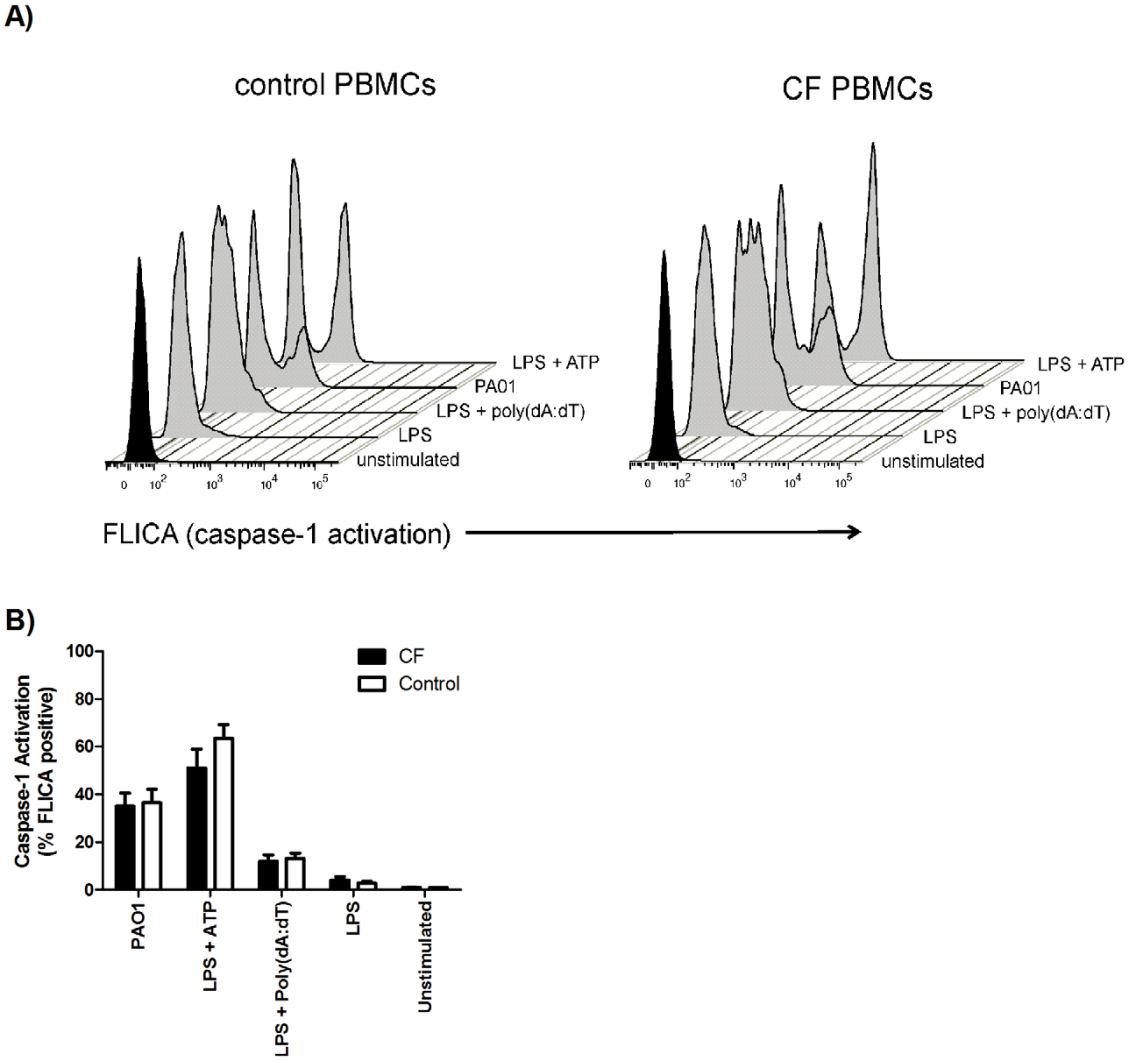
PBMCs from CF patients and controls show similar increases in caspase-1 activity upon inflammasome activation. PBMCs from CF patients (n = 6) and healthy controls (n = 6) were primed with LPS (10 ng/ml) for 5 hours prior to stimulation with ATP (5 mM) for 1 hour or Poly(dA:dT) (1 µg/ml) for 3 hours. PBMCs were stimulated with *P. aeruginosa* strain PAO1 for 3 hours. A representative histogram of the % caspase-1-active cells is shown in (A) with the averaged values shown in (B).

Monocytes were identified in PBMC populations using CD14 as a phenotyping marker. CD14 positive monocytes from CF patients and healthy controls showed a significant increase in caspase-1 activation upon stimulation with LPS+ATP, PAO1, and LPS+Poly(dA:dT) (Fig. 4a) but this activation was not different between CF and control subjects (Fig. 4b).

### PBMCs from CF patients do not produce increased amounts of IL-1β upon inflammasome stimulation

**Figure 5:**
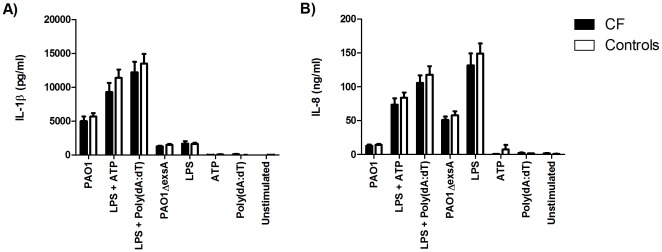
PBMCs from CF patients do not produce increased amounts of IL-1β. PBMCs from CF patients (n = 17–20) and healthy controls (n = 15–19) were primed with LPS (10 ng/ml) overnight and stimulated with *P. aeruginosa* PAO1 (MOI = 1), *P. aeruginosa* PAO1 lacking *exsA* (MOI = 1), ATP (5 mM), or Poly(dA:dT) (1 µg/ml) for 24 hours. *P. aeruginosa* lacking *exsA* was used as a type III secretion control in comparison with wild-type *P. aeruginosa*. Supernatants were assayed for (A) IL-1β and (B) IL-8.

Previous studies have shown that the loss of CFTR results in increased NF-κB activity and pro-inflammatory cytokine secretion [4], [5], [32], [36], [37]. To further examine this relationship, PBMCs from CF patients and healthy adult controls were stimulated with PAO1, LPS+ATP, and LPS+Poly(dA:dT), to activate the NLRC4, NLRP3, and AIM2 inflammasomes, respectively. By 24 hours of stimulation, CF PBMCs did not produce increased amounts of IL-1β (Fig. 5a) or IL-8 (Fig. 5b) when compared to healthy controls. However, we did notice a transient decrease (P<0.001) in the amount of IL-1β produced by CF cells in response to LPS+ATP at 6 hours (data not shown). Stimulation of PBMCs with *P. aeruginosa* that lacks *exsA* (PAO1Δ*exsA*), a key regulator of type III secretion, produced three-fold less IL-1β compared to the parental PAO1 strain by 24 hours (Fig. 5a). Inflammasome stimulation without priming did not result in any IL-1β production in either CF or control PBMCs. Contrary to our hypothesis, these results indicate that PBMCs from CF patients do not display increased production of IL-1β or IL-8 with inflammasome activation nor do they suggest any increased basal or induced NF-κB activity. These results are consistent with our observation that caspase-1 activity is not different between CF and control PBMCs (Fig. 4).

### NF-κB activation is required for IL-1β and IL-8 responses to *P. aeruginosa*

**Figure 6:**
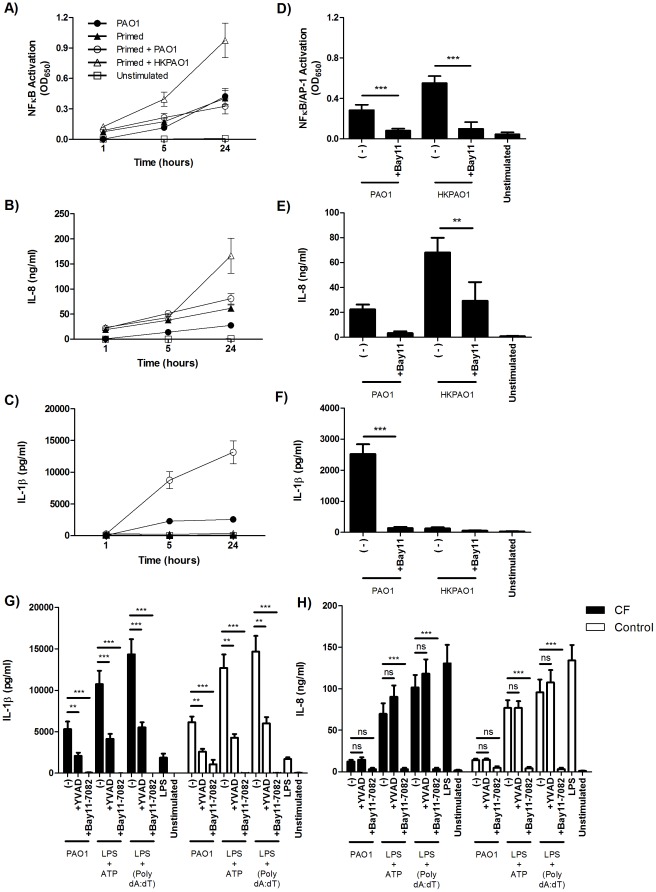
NF-κB activation potentiates the degree of IL-1β production and secretion upon inflammasome activation. THP-1 reporter cells were primed overnight with heat-killed *P. aeruginosa* and stimulated the next day with live *P. aeruginosa* or additional heat-killed *P. aeruginosa* for the times indicated. Cell culture supernatants were assayed for (A) NF-κB/AP-1 activity, (B) IL-8, and (C) IL-1β secretion (n = 3–6 experiments). Using the same stimulation method, THP-1 reporter cells were treated with Bay11-7082 (20 µM) for 1 hour prior to priming with heat-killed PAO1 or live PAO1. Supernatants were assayed at 24 hours for (D) NF-κB/AP-1 activity, (E) IL-8, and (F) IL-1β secretion (n = 3–5). PBMCs from CF patients (n = 11–15) and controls (n = 10–13) were treated with z-YVAD-fmk (20 µM) or Bay11-7082 (10 µM) and stimulated with live PAO1 (MOI = 1), ATP (5 mM), or Poly(dA:dT) (1 µg/ml) according to the schedule in Figure 1. (G) IL-1β and (H) IL-8 levels were measured at 24 hours. Statistical analysis was performed using two way ANOVA with Bonferroni correction for multiple comparisons. *, **, and *** signify P<0.05, 0.01, and 0.001.

We confirmed the dependence of PAO1-induced IL-1β and IL-8 production on NF-κB activation using THP-1 cells expressing a reporter driven by NF-κB and AP-1 response elements. We found that stimulation of primed THP-1 reporter cells with heat-killed PAO1 produced the highest levels of NF-κB/AP-1 activation (Fig. 6a) and this correlated with IL-8 secretion (Fig. 6b) but negligible amounts of IL-1β were secreted (Fig. 6c). Stimulation of primed THP-1 reporter cells with live PAO1 did not significantly increase NF-κB/AP-1 activity (Fig. 6a) or IL-8 secretion (Fig. 6b) over priming alone. However, IL-1β production was greatly augmented over primed cells stimulated with heat-killed PAO1 or unprimed cells stimulated with live PAO1 (Fig. 6c). This confirmed that NF-κB activation alone is not sufficient for maximal IL-1β secretion, but increased priming of NF-κB is capable of augmenting IL-1β production and secretion upon inflammasome stimulation. Dependency of these responses on NF-κB was confirmed by pharmacologic inhibition of NF-κB using the Bay11-7082 inhibitor of IκBα phosphorylation, which significantly reduced NF-κB/AP-1 activation (P<0.001) (Fig. 6d) and the subsequent production of IL-8 (P<0.01) (Fig. 6e) and IL-1β (P<0.001) (Fig. 6f) in response to both heat-killed and live PAO1. These results were also verified in CF and control PBMCs for each inflammasome examined (P<0.001) (Fig. 6g–h). Overall these results confirm that NF-κB is an important modulator of IL-1β production and that increased activation of NF-κB augments inflammasome-mediated production of IL-1β.

### Bay11-7082 inhibits pro-IL-1β production in response to *P. aeruginosa*

**Figure 7:**
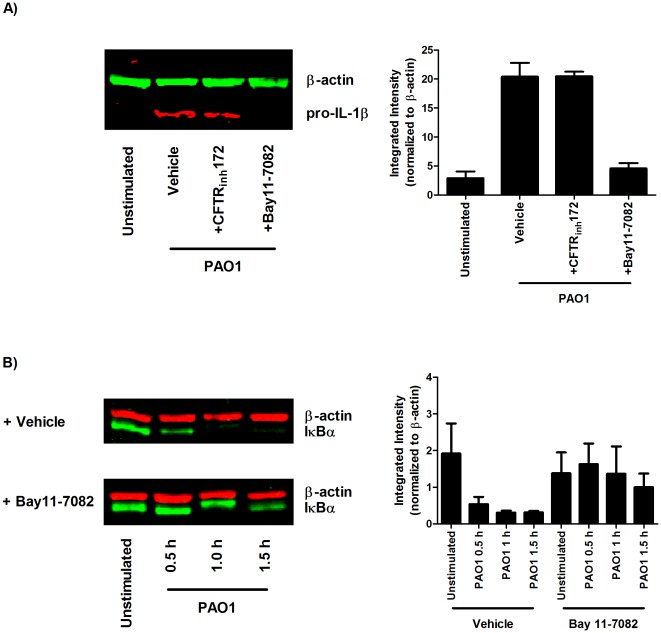
Bay11-7082 inhibits pro-IL-1β production in response to *P. aeruginosa*. PMA-differentiated THP-1 cells were treated with 10 µM CFTR_inh_172 or 20 µM Bay11-7082 and harvested after (A) 4 hours (n = 3) or (B) 0.5, 1, and 1.5 hours (n = 3) stimulation with PAO1. One representative blot is shown with a graph of the averaged fluorescence intensity values over 3 experiments.

In addition to inhibition of NF-κB activity, Bay11-7082 can also directly inhibit the NLRP3 inflammasome [38]. To validate its use in this study as an NF-κB inhibitor, western blots for pro-IL-1β were performed alongside inhibition of CFTR activity by CFTR_inh_172 in response to PAO1 at 4 hours after stimulation (Fig. 7a). Our results indicate that Bay11-7082 prevents production of pro-IL-1β whereas CFTR_inh_172 does not seem to affect it. This was further corroborated by the ability Bay11-7082 to inhibit IκBα degradation at 0.5, 1, and 1.5 hours post PAO1 stimulation (Fig. 7b).

### Disruption of CFTR activity does not increase IL-1β production in PBMCs and THP-1 cells

**Figure 8:**
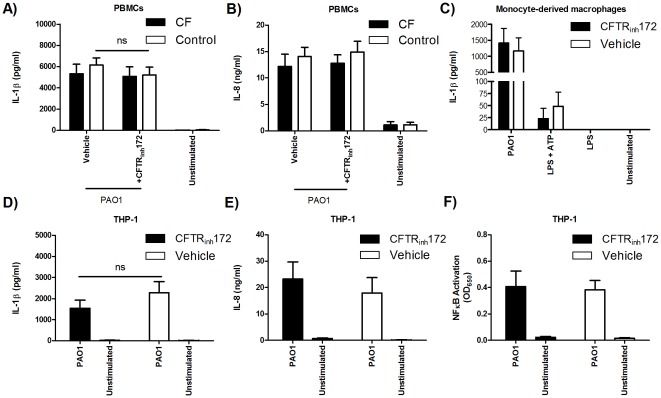
Disruption of CFTR activity does not increase IL-1β production in PBMCs or macrophages. PBMCs from CF patients (n = 15) and controls (n = 13) were treated with CFTR_inh_172 (10 µM) for 18 hours prior to stimulation with live PAO1 (MOI = 1). (A) IL-1β and (B) IL-8 production was measured at 24 hours. (C) Monocytes from controls (n = 3) were differentiated into macrophages. Macrophages were treated with CFTR_inh_172, stimulated as per monocytes, and measured for IL-1β production at 24 hours. THP-1 reporter cells were treated with CFTR_inh_172 24 hours prior to stimulation with PAO1 and measured for (D) IL-1β secretion, (E) IL-8, and (F) NF-κB/AP-1 activity at 24 hours (n = 4).

A previous study has indicated a role for chloride ion concentration in suppression of NLRP3 inflammasome activation [34]. To determine whether CFTR dysfunction alters IL-1β production, THP-1 cells and PBMCs from CF patients and healthy controls were treated with the CFTR inhibitor, CFTR_inh_172, prior to simulation with live *P. aeruginosa*. Treatment with CFTR_inh_172 did not alter IL-1β or IL-8 production in control subjects or CF patients (Fig. 8a–b). Similarly, IL-1β production was not different in monocyte-derived macrophages or THP-1 reporter cells treated with CFTR_inh_172 (Fig. 8c–d). IL-8 (Fig. 8e) and NF-κB activity (Fig. 8f) were also unchanged in CFTR_inh_172-treated THP-1 reporter cells.

## Discussion

Levels of IL-1β are increased in the BALF of CF patients but the cellular source of this cytokine and its production in the context of targeted inflammasome activation are still unclear. We first studied airway epithelial cells due to their role in barrier function, proximity to infection, and ability to produce high levels of pro-inflammatory cytokines. However, we found that bronchial epithelial cells do not produce significant amounts of IL-1β and do not show a significant increase in caspase-1 activation in response to PAO1 and LPS+ATP, in comparison to hematopoeitic mononuclear cells. Hematopoeitically-derived cells, such as monocytes and macrophages, appear to be a principal source of IL-1β. CFTR is expressed in alveolar macrophages [32], [39] as well as in PBMCs at both the mRNA and protein level [40], [41], and its loss is frequently associated with an augmented inflammatory phenotype.

Despite our findings indicating that bronchial epithelial cells when grown *in vitro* are unlikely to be significantly involved in the direct production of IL-1β (Fig. 2a–d), others have shown their capacity to respond to alveolar macrophage-derived IL-1β and to amplify the inflammatory response through the induction of chemokines and recruitment of inflammatory effector cells [42]. This interaction may constitute a critical component to effective host defense and diminishing the capacity of host cells to respond to IL-1β may leave the host susceptible to infections by pathogens such as *P. aeruginosa* [43]. Conversely, overproduction of IL-1β can also play a key role in chronic inflammatory responses and cause damage to the lung parenchyma [44], [45].

Although we hypothesized that CF cells would secrete increased amounts of IL-1β, we found that IL-1β production in CF PBMCs was not increased upon inflammasome stimulation as compared to controls (Fig. 5a–b). This was in contrast to a previous study from our group, which showed increased IL-1β production by CF PBMCs in response to LPS [46], although this difference may be accounted for by technical issues in stimulation time and dose. Moreover, IL-1β production was not increased in CF PBMCs with inflammasome stimulation alone as would be anticipated if there were basal levels of NF-κB activation. Cells deficient in CFTR are thought to exhibit an increased basal level of NF-κB activity, which leads to increased pro-inflammatory cytokine production including an increased availability of pro-IL-1β for cleavage and secretion. This amplification of IL-1β secretion was shown by priming THP-1 monocytes and PBMCs with heat-killed *P. aeruginosa* or LPS prior to stimulation with live *P. aeruginosa*. This dramatically increased IL-1β secretion over stimulation with live *P. aeruginosa* without priming (Fig. 6c). Similarly, if CF PBMCs expressed increased basal NF-κB activity, there would be an increase in IL-1β secretion in the absence of LPS priming. However, no increase in IL-1β was observed under basal or primed conditions. Studies investigating the production of IL-1β have been somewhat inconsistent. A study by Reininger *et al.* [43] provided evidence that human bronchial epithelial cells possessing the ΔF508 CFTR mutation had a slightly reduced capacity to produce IL-1β and lacked the ability to induce an early NF-κB activation in response to *P. aeruginosa*. Conversely, a study by Kotrange *et al.* [31] found that murine bone marrow-derived macrophages expressing ΔF508-CFTR produced increased amounts of IL-1β when compared to macrophages expressing normal CFTR in response to *Burkholderia cenocepacia* K56-2. The differentiation of monocytes into macrophages may partly account for the differences observed with the study by Kotrange. Inflammasome-mediated IL-1β production by monocytes and PBMCs does differ from macrophages [47], [48], [49] and macrophages are found to have higher expression of CFTR over monocytes [50]. However, as monocytes and other PBMCs express CFTR [50], [51] and produce large amounts of IL-1β, they are adequate models to examine the effects of CFTR function on IL-1β production. Other hematopoeitic cells may also contribute to IL-1β production. For example, neutrophil counts can be significantly increased in the lungs of CF patients [52], [53], [54] and may produce mature IL-1β through caspase-1 independent mechanisms [55].

The role of NF-κB activation in inflammasome activation and IL-1β secretion is not straightforward. Studies have revealed an essential role for NF-κB activation in the production of pro-IL-1β and inflammasome components such as NLRP3 [22], [23]. In contrast, deletion of IKKβ, a kinase essential in NF-κB activation, increases IL-1β secretion in murine macrophages [56], [57] and demonstrates a dual role for NF-κB in regulation of IL-1β. To address this uncertainty in our experiments we also quantified IL-8, an important CF cytokine and marker of NF-κB activation [58], and found no differences between CF and control subjects. Similarly, levels of intracellular pro-IL-1β in THP-1 cells were dependent on NF-κB activity and did not increase with CFTR_inh_172 treatment. Subsequent treatment of THP-1 cells and PBMCs with the NF-κB inhibitor Bay11-7082 significantly inhibited both IL-1β and IL-8 secretion (Fig. 6d–h). Therefore, the IL-1β and IL-8 responses observed were both dependent upon NF-κB activation. Priming with heat-killed *P. aeruginosa*, like LPS, is unable to induce a strong IL-1β response as compared to live *P. aeruginosa* (Fig. 6c), but generated greater NF-κB/AP-1 activation (Fig. 6a) and IL-8 secretion (Fig. 6b) than live bacteria despite stimulation at equivalent MOIs. This may be indicative of the different degree and quality of the inflammatory response generated by live as opposed to dead bacteria [59].

Potential shortcomings of these experiments include its translatability to lung disease and issues related to the hypermutability of *P. aeruginosa* during the evolution of chronic infection. Although the responses measured in peripheral blood cells may not completely reflect the responses occurring in the CF lung, PBMCs have a number of useful advantages: (i) PBMCs are not subject to alterations that may emerge from long-term cell culture, cloning and immortalization, and (ii) PBMCs express a large repertoire of innate immune receptors and secrete a broad array of cytokines and chemokines allowing comprehensive analysis of the modulation of inflammatory responses by CFTR. Consideration must also be given to the nature of *P. aeruginosa* infection and genotypic changes in *P. aeruginosa* as infection progresses. *P. aeruginosa* mediates inflammasome activation through its type III secretion system (T3SS) and the NLRC4 inflammasome [19], [20]. However, clones of *P. aeruginosa* established during chronic infection may accumulate mutations in virulence factors such as *exsA* [60]. By employing a deletion mutant in *exsA*, the key regulator in T3SS transcription, we confirmed that the T3SS is important for IL-1β secretion (Fig. 5a–b), and that depending on the adaptation in type III secretion, the host IL-1β response may be up or downregulated [19], [60], [61].

In conclusion, our data are consistent with a role for hematopoietic cells, not airway epithelial cells, as the major source of inflammasome-mediated IL-1β production in the lungs in response to ATP and *P. aeruginosa*. Furthermore, we find little evidence to support an increased IL-1β inflammatory response to NF-κB/Inflammasome stimulation in CF patients. Further studies are warranted to determine if adaptations of *P. aeruginosa* during the course of chronic lung infection alters inflammasome activation, and whether this can be correlated with disease severity in CF.

## Materials and Methods

### Ethics Statement

Blood samples were obtained with informed written consent from control subjects and CF patients at the BC Children's Hospital. Consent was obtained for children by their parent or legal guardian. Subjects 7 years of age and older were required to provide informed assent as well. Protocols were approved by the Clinical Research Ethics Board (H09-01192).

### Cell Culture

CF (IB3-1 and CuFi-1) and control (S9 and NuLi-1) cells were obtained from the American Type Culture Collection. IB3-1 cells were derived from a patient expressing the ΔF508 and W1282X mutations and CuFi-1 were derived from a ΔF508 homozygous patient. S9 cells are IB3-1 cells that have been transfected with CFTR using an adeno-associated viral vector and NuLi-1 cells were derived from a patient possessing a wild-type CFTR genotype. THP1-XBlue cells stably express a secreted embryonic alkaline phosphatase (SEAP) reporter inducible by NF-κB and AP-1 (Invivogen). Cells were cultured as recommended by their respective suppliers using standard protocols. S9 and IB3-1 cells were cultured in basal LHC-8 (Invitrogen) supplemented with 10% (v/v) fetal bovine serum (FBS), 2 mM L-glutamine, and 1 mM sodium pyruvate. NuLi-1/CuFi-1 cells were cultured in BEBM serum-free medium (Lonza) with supplement bullet kit (EGF, hydrocortisone, bovine pituitary extract, transferrin, bovine insulin, triiodothyronine, epinephrine, retinoic acid), 2 mM L-glutamine, and 1 mM sodium pyruvate. PBMCs from CF patients and controls were cultured in RPMI-1640 (Hyclone) supplemented with 10% FBS, 2 mM L-glutamine, and 1 mM sodium pyruvate (complete RPMI). THP1-XBlue cells were cultured in complete RPMI with the addition of zeocin (100 µg/ml) to select for cells expressing the SEAP NF-κB/AP-1 reporter. Prior to stimulation, bronchial epithelial cell lines were plated in coated [62] 96-well plates (BD Biosciences) at 3×10^4^ cells/well unless indicated, and allowed to adhere overnight. Plates for S9 and IB3-1 stimulations were coated in a mixture of bovine serum albumin (100 µg/ml), fibronectin (10 µg/ml), and bovine collage type I (30 µg/ml) (BD Biosciences). Plates for NuLi-1 and CuFi-1 were coated with collagen type IV (60 µg/ml) (Sigma Aldrich). PBMCs were plated in 96-well plates at a density of 1.5×10^5^ cells/well in 200 µl (7.5×10^5^ cells/mL). THP-1 reporter cells were differentiated into a macrophage-like phenotype using 50 ng/ml of phorbol 12-myristate 13-acetate (PMA) (Sigma Aldrich) for 24 hours at a density of 1×10^5^ cells/well in 200 µl (5×10^5^ cells/ml). Cells were washed with PBS and allowed to rest a further 42 hours prior to stimulation.

### CF and control subject PBMCs

The diagnosis of CF was established by typical clinical features, increased sweat chloride concentrations (>60 mmol/l), and detection of CF-inducing mutations. All patients with CF were clinically stable at the time of blood donation, and we excluded any subjects who were receiving systemic corticosteroids due to potential immunomodulatory activity. Control samples were provided by healthy adult volunteers. In previously published work we have demonstrated that TLR-mediated inflammatory responses are stable in humans from birth to 60 years old [63], therefore did not age-match the CF patients and control subjects. Peripheral blood was collected in sodium heparin tubes (BD Biosciences) and PBMCs were isolated using density gradient centrifugation on Ficoll-Paque™ Plus (GE Healthcare). The layer containing PBMCs was isolated, washed twice in PBS and resuspended in complete RPMI. Cells were enumerated by trypan blue exclusion using the Countess automated cell counter (Invitrogen). For derivation of macrophages from monocytes, monocytes were allowed to adhere to plastic for 2 hours in RPMI 1640 after which non-adherent cells were removed. Monocytes were allowed to differentiate in RPMI 1640 supplemented with 10% human AB serum for 10 days.

### Cell stimulation and cytokine quantification

Bronchial epithelial cells were plated and allowed to adhere overnight prior to stimulation. Bronchial epithelial cells were rested or primed with LPS for 5 hours and stimulated with live *P. aeruginosa* PAO1 or ATP for the times indicated. PBMCs and THP-1 reporter cells were either rested or primed with LPS (Invivogen) or heat-killed PAO1 overnight (16 hours). The next day the cells were challenged with live PAO1, PAO1Δ*exsA*, ATP (Invivogen), or Poly(dA:dT) (Sigma Aldrich) for the times indicated (see Fig. 1). For stimulations with Poly(dA:dT), lipofectamine LTX was used at a 1∶1 (w∶v) ratio of µg of DNA to µl of lipofectamine and was mixed 30 minutes prior to stimulation. The NF-κB inhibitor Bay11-7082 (Invivogen) was added to cultures 1 hour prior to priming. If no priming was involved, inhibitor was added 1 hour prior to inflammasome stimulation. The CFTR inhibitor CFTR_inh_172 (Sigma Aldrich) was added to cultures 18 hours prior to inflammasome stimulation. The caspase-1 inhibitor z-YVAD-fmk (Biovision) was added to cultures 1 hour prior to inflammasome stimulation. Supernatants were collected and stored at −20°C. Cytokines released into supernatants from PBMCs stimulated with inflammasome activators were quantified using sandwich ELISA (eBioscience).

### Immunoblotting

1×10^6^ cells were seeded in 12-well plates, stimulated as indicated, and lysed in RIPA buffer supplemented with Halt protease and phosphatase inhibitor cocktail (Thermo Scientific). Protein concentrations were determined by Bradford assay (Thermo Scientific). Lysates were resolved by electrophoreses on 10% SDS-polyacrylamide gels and transferred onto PVDF membranes (Millipore). Blots were blocked for 1 hour at room temperature and probed overnight at 4°C for pro-IL-1β (Santa Cruz), IκBα (Cell Signaling), or β-actin (Cell Signaling). Blots were subsequently probed with fluorescently-labeled secondary antibodies, IRDye® 680 or 800CW (LI-COR Biosciences) for 1 hour. Both blocking and probing steps were carried out in tris-buffered saline (G Biosciences) containing 5% bovine serum albumin and 0.1% TWEEN 20 (Calbiochem). Blots were imaged on a LI-COR Odyssey infrared imaging system (LI-COR Biosciences) and quantified using the included analysis software.

### Quantification of caspase-1 activity

Bronchial epithelial cells were plated in 6-well plates at 5×10^5^ cells/well overnight. Cells were primed with LPS for 5 hours and stimulated with ATP for 1 hour or stimulated with live PAO1 for 3 hours. PBMCs were stimulated the same day as blood donation. PBMCs were seeded in a 96-well plate at a density of 4.5×10^5^ cells/well (2.5×10^6^ cells/ml), primed with LPS for 5 hours and then stimulated with ATP for 1 hour or Poly(dA:dT) for 3 hours or stimulated with live PAO1 for 3 hours. Caspase-1 activity was measured using FLICA (Immunochemistry Technologies), a cell-permeable fluorescent probe (FAM-YVAD-fmk) that binds active caspase-1. Cells were incubated 1 hour with FLICA at 37°C and stained with PE-Cy7-conjugated anti-CD14 antibodies (eBioscience) to identify monocytes. The gating strategy consisted of including live cells that were CD14 positive which were subsequently analyzed for the frequency of FLICA positive cells.

### NF-κB/AP-1 Activity Assay

Supernatants from THP-1 reporter cells were incubated with Quanti-Blue substrate (Invivogen) at 37°C and allowed to develop for 16–18 hours. Quanti-Blue contains a substrate for alkaline phosphatase and changes in the amount of NF-κB/AP-1 activity were quantified by optical density (λ = 655) measured using a SpectraMax 384 Plus plate reader and SoftMax Pro software (Molecular Devices).

### Bacterial strains

*P. aeruginosa* laboratory strains PAO1 and the PAO1Δ*exsA* mutant were obtained from Dr. Robert Hancock. *P. aeruginosa* strains PAO1 and PAO1Δ*exsA* were grown from overnight cultures in Luria Bertani (LB) broth and LB+streptomycin (150 µg/ml) until mid-logarithmic phase. Cells were washed once in PBS and resuspended in PBS to an optical density of 0.5 (λ = 600 nm). To prepare heat-killed bacteria, live PAO1 was resuspended in PBS to an optical density of 0.5 and heated at 60°C for 1 hour. For stimulations, live PAO1 was resuspended to an optical density of 0.5 in PBS and further diluted in culture medium prior to stimulation to achieve the desired multiplicity of infection. Heat-killed PAO1 was added in a volume equivalent to that used to achieve an MOI of 1 for live PAO1.

### Statistics

All graphs display the mean ± SEM and were generated with Prism 5 (Graphpad). Statistical significance was determined by performing one or two-way ANOVA and the Bonferroni post-test where applicable.
